# Novel Insights into Pathophysiology of Delayed Cerebral Ischemia: Effects of Current Rescue Therapy on Microvascular Perfusion Heterogeneity

**DOI:** 10.3390/biomedicines11102624

**Published:** 2023-09-24

**Authors:** Björn B. Hofmann, Cihat Karadag, Christian Rubbert, Simon Schieferdecker, Milad Neyazi, Yousef Abusabha, Igor Fischer, Hieronymus D. Boogaarts, Sajjad Muhammad, Kerim Beseoglu, Daniel Hänggi, Bernd Turowski, Marcel A. Kamp, Jan F. Cornelius

**Affiliations:** 1 Department of Neurosurgery, Medical Faculty and University Hospital Düsseldorf, Heinrich-Heine-University Düsseldorf, 40225 Düsseldorf, Germany; 2 Department of Diagnostic and Interventional Radiology, Medical Faculty and University Hospital Düsseldorf, Heinrich-Heine-University Düsseldorf, 40225 Düsseldorf, Germany; 3 Department of Neurosurgery, Medical Faculty, Radboud University Nijmegen, 6525 GA Nijmegen, The Netherlands; 4 Department of Neurosurgery, International Neuroscience Institute, 30625 Hannover, Germany; 5 Centre for Palliative and Neuropalliative Care, Brandenburg Medical School Theodor Fontane, Campus Rüdersdorf, 15562 Rüdersdorf bei Berlin, Germany; 6 Faculty of Health Sciences Brandenburg, Brandenburg Medical School Theodor Fontane, 16816 Neuruppin, Germany

**Keywords:** aneurysmal subarachnoid haemorrhage, delayed cerebral ischemia, intra-arterial nimodipine, microvascular perfusion heterogeneity, rescue therapy, spasmolysis

## Abstract

General microvascular perfusion and its heterogeneity are pathophysiological features of delayed cerebral ischemia (DCI) that are gaining increasing attention. Recently, CT perfusion (CTP) imaging has made it possible to evaluate them radiologically using mean transit time (MTT) and its heterogeneity (measured by cvMTT). This study evaluates the effect of multimodal rescue therapy (intra-arterial nimodipine administration and elevation of blood pressure) on MTT and cvMTT during DCI in aneurysmal subarachnoid haemorrhage (aSAH) patients. A total of seventy-nine aSAH patients who underwent multimodal rescue therapy between May 2012 and December 2019 were retrospectively included in this study. CTP-based perfusion impairment (MTT and cvMTT) on the day of DCI diagnosis was compared with follow-up CTP after initiation of combined multimodal therapy. The mean MTT was significantly reduced in the follow-up CTP compared to the first CTP (3.7 ± 0.7 s vs. 3.3 ± 0.6 s; *p* < 0.0001). However, no significant reduction of cvMTT was observed (0.16 ± 0.06 vs. 0.15 ± 0.06; *p* = 0.44). Mean arterial pressure was significantly increased between follow-up and first CTP (98 ± 17 mmHg vs. 104 ± 15 mmHg; *p* < 0.0001). The combined multimodal rescue therapy was effective in addressing the general microvascular perfusion impairment but did not affect the mechanisms underlying microvascular perfusion heterogeneity. This highlights the need for research into new therapeutic approaches that also target these pathophysiological mechanisms of DCI.

## 1. Introduction

Not only the initial bleeding event in aneurysmal subarachnoid haemorrhage (aSAH) but also the subsequent pathophysiological processes lead to the high mortality and morbidity associated with this disease [1,2,3]. Patients may suffer a secondary deterioration of their neurological condition, called delayed cerebral ischaemia (DCI), which can significantly impact the outcome [1,4,5]. Ecker and Riemenschneider first observed arterial narrowing following aSAH in 1951 [6]. In the following decades, macro-vasospasms were believed to result in cerebral hypoperfusion, infarctions, neurological deterioration, and poor outcomes [6,7,8]. Subsequently, most therapeutic approaches and clinical randomised controlled trials (RCT) aimed to eliminate macro-vasospasms to improve the outcome [9,10,11,12,13,14,15]. Common examples are nimodipine RCTs [11,12,13] or local intra-arterial administration of nimodipine as a rescue therapy of vasospasms [14,15]. However, as demonstrated in the CONSCIOUS trials, effective treatment of macro-vasospasms per se does not translate to more favourable outcomes [10,16,17]. These results meant that cerebral macro-vasospasm was not the sole cause of late neurological deterioration and disastrous outcomes following aSAH. Moreover, DCI is characterised by a potpourri of various pathophysiological dysfunctions [18,19,20,21]. Capillary transit time heterogeneity (CTH) as one pathophysiological mechanism of DCI represents a new but, in our view, highly relevant pathophysiological concept. CTH extends the classical flow-diffusion equation and states that cerebral oxygenation depends not only on total net cerebral blood flow (CBF) but also on capillary and maybe also microvascular flow distribution, which is also affected in aSAH by a wide variety of pathophysiological mechanisms such as microvascular dysfunction, inflammation, or microthrombosis [18,19,20,21]. Recently, heterogeneity of the mean transit time (cvMTT) in CT perfusion imaging, as a radiological measurement of microvascular perfusion heterogeneity [22], was demonstrated to be correlated with poor neurological outcomes in aSAH patients in the DCI phase [23].

Similar to the pathophysiological concepts behind DCI, therapeutic concepts have also changed. For a long time, the so-called “triple H” therapy consisting of hypertension, hypervolemia, and haemodilution was the treatment of choice for cerebral vasospasm and DCI [24,25,26,27]. In many innovative neurovascular centres, severe DCI is now treated multimodally, mainly by induced hypertension, careful monitoring, and, when needed, administration of local intra-arterial nimodipine (spasmolysis) as rescue therapy [28,29]. Under effective multimodal therapy, mechanical treatment of hemodynamically relevant macro-vasospasm may become a relatively rare procedure.

However, the therapeutical effects of this rescue therapy on microvascular perfusion heterogeneity in patients suffering from DCI are currently unclear. In the present study, we therefore investigated the effects of multimodal rescue therapy, including blood pressure elevation and intra-arterial nimodipine administration in the DCI phase, on microvascular perfusion heterogeneity, as measured by the cvMTT of CT perfusion imaging, in aSAH patients.

## 2. Materials and Methods

All procedures performed in studies involving human participants were in accordance with the ethical standards of the institutional committee and with the 1964 Helsinki Declaration and its later amendments. This study was approved by the local ethics committee (study ID: 5760R) and the need for written informed consent was waived. Data will be made available upon reasonable request. We prepared the manuscript according to the Strengthening the Reporting of Observational Studies in Epidemiology (STROBE) guidelines [30] and the EQUATOR Network recommendations for preparing scientific manuscripts.

### 2.1. Inclusion and Exclusion Criteria

We retrospectively included all patients with SAH admitted to our tertiary-care hospital between May 2012 and December 2019 who met the following inclusion criteria: (1) patient treated for SAH, (2) the treatment was performed in our specialized neurosurgical intensive care unit, (3) the patient received CTP imaging on two consecutive days due to MTT increase in the first imaging, and (4) the patient received an intra-arterial administration of nimodipine (spasmolysis) during her or his stay in the intensive care unit. Patients were excluded from the study on the basis of the following criteria: (1) there was no CTP before the spasmolysis (on the same day) or (2) no CTP on the day following the spasmolysis was available; if (3) the image quality of a CTP imaging was insufficient for the evaluation; or (4) if there was a definite cause for subarachnoid haemorrhage other than a ruptured aneurysm (e.g., bleeding after an operation).

### 2.2. aSAH Management

We managed patients according to an in-house standardised protocol for general care [31] based on the applicable aSAH guidelines [32]. In brief, we aim for a surgical or endovascular occlusion of the aneurysm within 24 h after admission. If there is no reason for immediate surgical treatment (e.g., need for intraparenchymatous haemorrhage evacuation), patients are monitored in our ICU with the “no-touch technique” until digital subtraction angiography is performed. Patients with a Glasgow Coma Scale ≤ 12 were sedated, intubated, and ventilated due to the limited neurological accessibility. External ventricular drainage (EVD) was performed in all intubated patients. Once sedation was started, it was not discontinued before the aneurysm was occluded. Systolic arterial blood pressure was adjusted between 100 and 140 mmHg in all patients, and the partial pressure of carbon dioxide (pCO2) was maintained between 30 and 35 mmHg for all intubated patients. After occlusion of the aneurysm, we generally aim to end sedation with the goal of extubation as soon as possible, unless there are contra-indications (for example, uncontrollable generalised seizures or intracranial pressure that cannot be controlled by cerebrospinal fluid drainage). During the treatment period, patients received systemic nimodipine for at least one week after bleeding. In the initial phase, nimodipine was administered intravenously with a steady rate of 2 mg/h, while in the later treatment, oral administration of 60 mg nimodipine every 4 h was conducted. If high rates of intravenous catecholamines were needed to sustain target blood pressure, the systemic nimodipine dose may have been reduced.

### 2.3. Timing of Perfusion CT

After aneurysm repair, CTP imaging followed our local DCI diagnosis and treatment guidelines [31]. We performed CTP imaging every 3 days starting 6 h after the aneurysm occlusion. The last routine CTP imaging was performed on the 13th or 14th day. Additional CTP imaging is pursued in acute cases when a patient shows neurological deterioration without any other cause at any time during treatment.

### 2.4. Multimodal Rescue Therapy

Based on CTP imaging, especially MTT prolongation, and clinical symptoms, further therapy was decided upon in an interdisciplinary consultation. If there was a clinical correlate, i.e., a neurological deterioration without any other reason or MTT prolongation in accordance with the DCI criteria [33], a total dose of 3.2 mg nimodipine was administered intra-arterially in both Internal Carotid Arteries (ICA), regardless of lateralisation of the CTP alterations. Two injections of 0.8 mg nimodipine were given sequentially, each over 5 min, through a 5F diagnostic catheter placed in each ICA. A control angiogram was performed 5 min after each injection. Furthermore, a sustained increase in blood pressure in the intensive care unit was carried out within the frame of the patient’s individual possibilities (usually with the help of intravenous Noradrenaline in individualised doses). After induction of the multimodal rescue therapy as just described, the patient underwent CTP imaging on the following day (approx. 24 h after induction) to monitor the effect of the therapy.

### 2.5. Analysis of Perfusion CT Imaging

We acquired CTP data using a multislice CT scanner (Somatom Volume Zoom, Definition Flash, or AS; Siemens Healthineers, Munich, Germany; 80 kV, 120 mAs, 2 adjacent slices, 10 mm slice thickness, 1 image/s over 35 s) as previously described [23]. Contrast-enhancing medium (30 mL, 400 mg iodine/mL) followed by a saline chaser (30 mL) was injected with a flow rate of 5 mL/s three seconds after starting the CT scan. Intravenous access of ≤18 gauge in a cubital vein or a high-flow central venous catheter was used for contrast administration. The slices were positioned at the level of the central parts of the lateral ventricles, parallel to a plane through the orbital floor and the external auditory meatus, enabling sampling of the anterior, middle, and posterior cerebral artery territories as well as the anterior and posterior borderzones. We used STROKETOOL-CT (Digital Image Solutions, Frechen, Germany) to calculate the parameter images (MTT, CBF, cerebral blood volume [CBV], and time-to-maximum [Tmax]), which processed data using singular value decomposition. We utilised Angiotux CT 2D (ECCET 2006, Beck A. Aurich V., Langenfeld, Germany, http://www.eccet.de/, accessed on 1 June 2023) for standardised parameter value extraction from the different cortical brain regions. Angiotux automatically delineated a 10 mm wide band along the cortex, excluding outer CSF spaces, the rostral falx cerebri, and the superior sagittal sinus. We reviewed the automated definition of the region of interest and corrected any potential deviations. A running average spanning 10° of the ROI was computed in 2° steps for each perfusion parameter, yielding 180 measurements per parameter per CTP scan.

### 2.6. Definition of Outcome Measures

We defined heterogeneity of microvascular perfusion as heterogeneity of cerebral perfusion among the measured ROIs, assessed by the coefficient of variation (cv or relative SD) of the MTT (cvMTT) for each individual scan. The cv is a measure of the dispersion of a probability distribution independent of the mean of the examined variable [23]:

$$\mathrm{cvMTT}=\frac{\mathrm{standard}\;\mathrm{deviation}}{\mathrm{expected}\;\mathrm{value}}=\frac{\mathrm{standard}\;\mathrm{deviation}\;\mathrm{MTT}}{\mathrm{mean}\;\mathrm{MTT}}$$

The analysis of this study included the CTP demonstrating the initial prolongation of the MTT and the control CTP on the following day after the induction of multimodal rescue therapy within the DCI period. If a patient received multiple endovascular rescue therapies in one stay, only the CTP pair of the first rescue therapy was considered in this study.

Demographic data including age at diagnosis and sex were collected retrospectively from patients’ charts. For further correlation with cvMTT, we selected the mean arterial blood pressure, the intracranial pressure (ICP), and the cerebral perfusion pressure (CPP), recorded as previously described in detail [34]. In brief, the last blood and intracranial pressure values recorded by the digital documentation system before or the first value after the transport to CT imaging were collected since no values were saved in the data system during the transport itself (50-metre transport distance to CT). The value with a shorter time interval to the actual imaging was preferred. If values were recorded with the same time interval, the values prior to transport were preferred.

### 2.7. Statistical Analysis

Continuous variables are presented as mean ± standard deviation, ordinal values are presented as median values and minimum–maximum ranges. For categorical data, frequencies and percentages are presented. For each patient, mean arterial pressure (MAP) and CPP were measured and average MTT was calculated from the CTP imaging, both before and after therapy. In addition, the coefficient of variation (cv) of the MTT values was calculated for each patient. Numerical values between two discrete groups were compared using the *t*-test, and correlations between two numerical variables were compared using linear regression analysis. Significance was defined as *p*-value < 0.05. All calculations were performed in Python 3.9.16, using numpy, scipy, and statsmodels libraries.

## 3. Results

### 3.1. Patient Cohort

**Figure 1:**
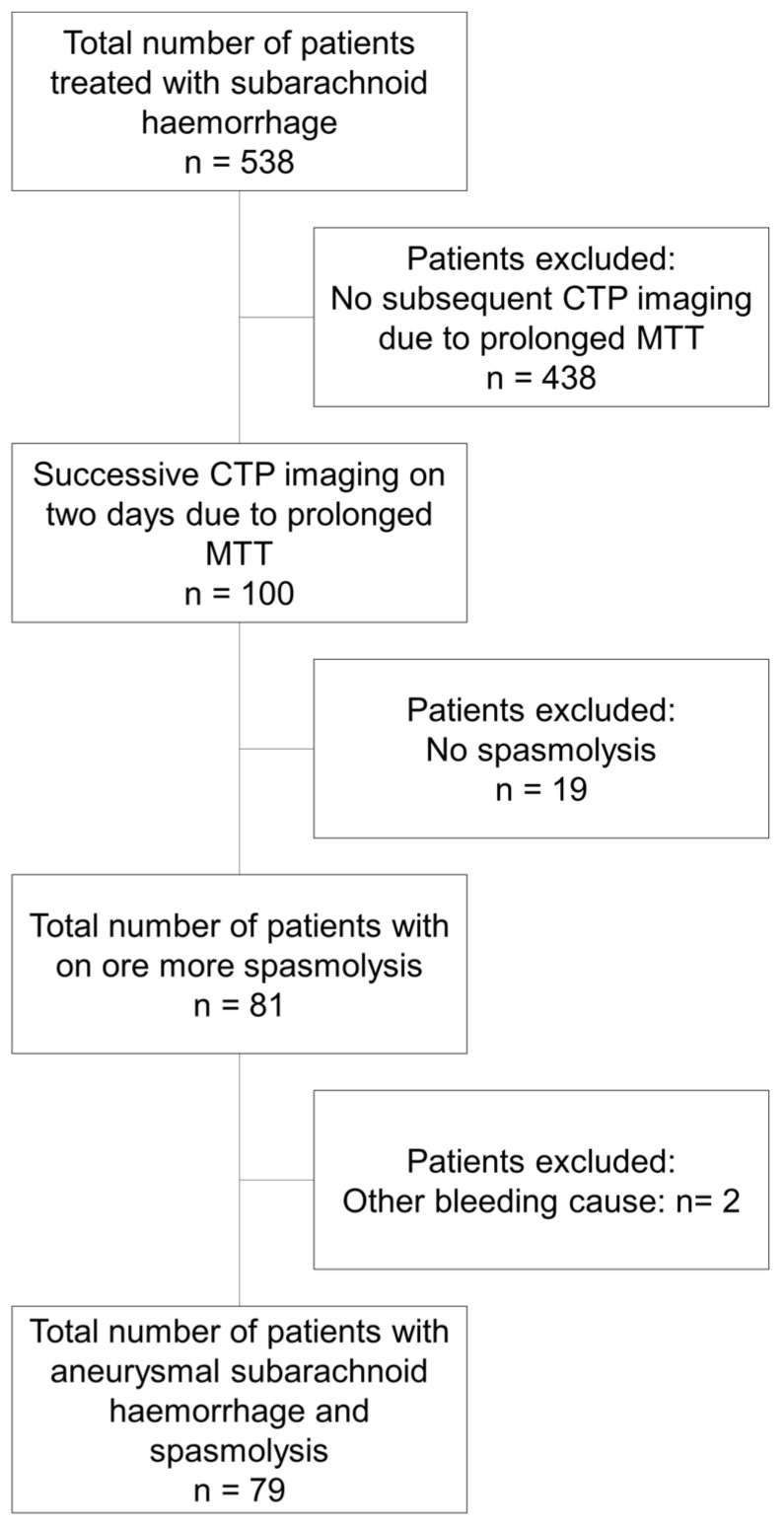
Flow chart describing the patient cohorts and selection process.

Seventy-nine of the initial five hundred and thirty-five patients with aSAH developed severe DCI requiring multimodal rescue therapy with intra-arterial nimodipine application during the observation period and were included in the analysis (Figure 1). The mean age was 55 ± 11 years and 72% (57) were women. Further patient characteristics are given in Table 1.

### 3.2. Influence of Multimodal Rescue Therapy on Blood Pressure and Cerebral Perfusion Pressure

**Figure 2:**
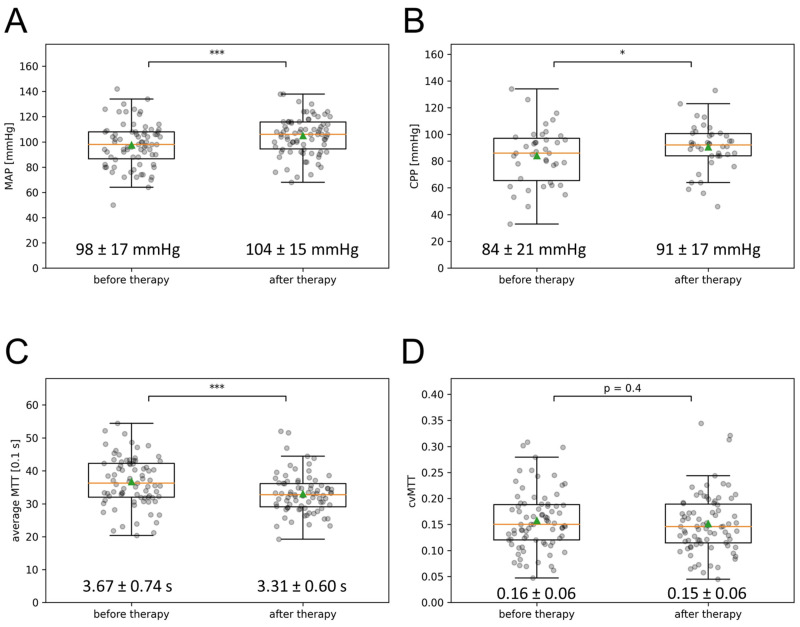
Effect of multimodal therapy on MAP, CPP, mean MTT, and cvMTT. (**A**) The MAP, (**B**) the CPP, (**C**) the mean MTT, and (**D**) the cvMTT at the time of the first and second CTP imaging are depicted in the box-and-whisker plots; boxes represent the interquartile range limited by the 25th and 75th percentile, the green triangle symbolizes the mean, the orange line the median, and the whiskers represent the maximum and minimum values. Values written out as mean ± SD (* *p* < 0.05; *** *p* < 0.001). CPP = cerebral perfusion pressure, cvMTT = coefficient of variation for MTT, MAP = mean arterial pressure, MTT = mean transit time, s = second.

The MAP at the first CTP imaging was 98 ± 17 mmHg and increased significantly after the introduction of the multimodal rescue therapy to 104 ± 15 mmHg at the time of the second CTP imaging 24 h after the induction of the multimodal rescue therapy (±SD; *p* < 0.0001), as depicted in Figure 2A. The CPP significantly increased from 84 ± 21 mmHg to 91 ± 17 mmHg in the subgroup of 39 patients with intracranial pressure measurement (±SD; *p* < 0.05) (Figure 2B).

### 3.3. Influence of Multimodal Rescue Therapy on Microvascular Perfusion Heterogeneity (cvMTT)

Contrary to the mean MTT, there was no significant change in the cvMTT (initial CTP: cvMTT = 0.16 ± 0.06; CTP 24 h after induction of multimodal rescue therapy: cvMTT = 0.15 ± 0.06; *p* = 0.44, Figure 2D).

### 3.4. Influence of Multimodal Rescue Therapy on General Microvascular Perfusion (mean MTT)

The mean MTT of the initial CTP imaging was 3.67 ± 0.74 s and decreased significantly to 3.31 ± 0.60 s in the follow-up CTP imaging after the initiation of the multimodal rescue therapy, as depicted in Figure 2C (*p* < 0.0001).

### 3.5. Correlation of MAP and CPP with mean MTT and cvMTT

**Figure 3:**
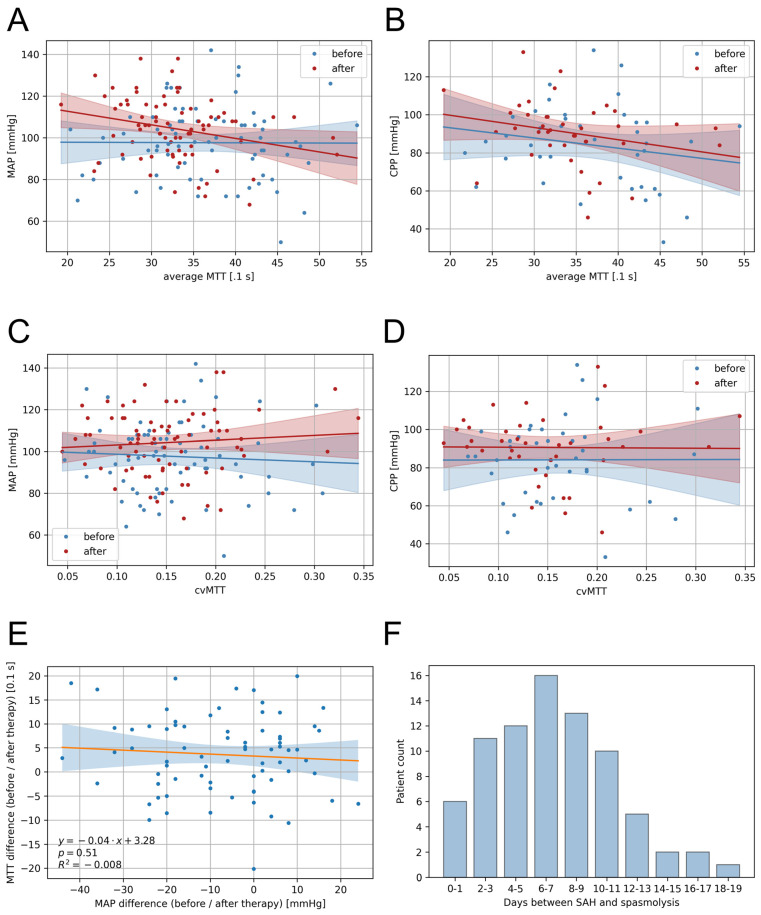
Correlations of the blood pressure and CT perfusion parameters and presentation of the temporal distribution. (**A**) The mean MTT showed no significant correlation with the MAP for the first CTP imaging (Dependency MAP ~ MTT CTP1: *p* = 0.96, R^2^ = −0.014) but a weak correlation for the second CTP imaging (Dependency MAP ~ MTT CTP2: *p* = 0.02, R^2^ = 0.054). (**B**) The mean MTT showed no significant correlation with the CPP for the first CTP imaging (Dependency CPP ~ MTT CTP1: *p* = 0.95, R^2^ = −0.026) and the second CTP imaging (Dependency CPP ~ MTT CTP2: *p* = 0.13, R^2^ = 0.035). (**C**) The cvMTT showed no significant correlation, neither with the MAP (Dependency MAP ~ cvMTT CTP1: *p* = 0.62, R^2^ = −0.010; Dependency MAP ~ cvMTT CTP2: *p* = 0.46, R^2^ = −0.006) nor (**D**) the CPP (Dependency CPP ~ cvMTT CTP1: *p* = 0.99, R^2^ = −0.026; Dependency CPP ~ cvMTT CTP2: *p* = 0.95, R^2^ = −0.026) at the first and the second CTP imaging. (**E**) Correlation of MTT and MAP differences between the first and the second CTP imaging (linear regression). (**F**) Number of days between the bleeding event and initiation of multimodal rescue therapy in the patient cohort shown as a bargraph. CPP = cerebral perfusion pressure, cvMTT = coefficient of variation for MTT, MAP = mean arterial pressure, MTT = mean transit time, R = regression coefficient.

Correlations of the MAP and CPP with the cerebral perfusion (mean MTT and cvMTT) were analysed using linear regression analysis. The mean MTT showed no significant correlation with the MAP before multimodal rescue therapy (dependency MAP ~ MTT CTP1: *p* = 0.96, R^2^ = −0.014; Figure 3A) and a weak correlation post multimodal rescue therapy (dependency MAP ~ MTT CTP2: *p* = 0.02, R^2^ = 0.054; Figure 3A). For patients with ICP measurements, the mean MTT showed no significant correlation with the CPP at any time (pre-multimodal rescue therapy: dependency CPP ~ MTT CTP1: *p* = 0.95, R^2^ = −0.026; post-multimodal rescue therapy; Figure 3B).

For the cvMTT, there was no significant correlation with either the MAP (Figure 3C; dependency MAP ~ cvMTT CTP1: *p* = 0.62, R^2^ = −0.010; dependency MAP ~ cvMTT CTP2: *p* = 0.46, R^2^ = −0.006) or the CPP (Figure 3D; dependency CPP ~ cvMTT CTP1: *p* = 0.99, R^2^ = −0.026; dependency CPP ~ cvMTT CTP2: *p* = 0.95, R^2^ = −0.026) at any time.

### 3.6. Correlation of MAP and MTT Changes Induced by Multimodal Rescue Therapy

Linear regression analysis showed no significant correlation between the change in MAP induced through multimodal rescue therapy and the mean MTT change between the first and the second CTP imaging (*p* = 0.51, R^2^ = −0.008) (Figure 3E).

### 3.7. Timing of Multimodal Therapy Initiation

Figure 3F shows the distribution of the days on which multimodal rescue therapy was initiated after the bleeding event.

## 4. Discussion

This retrospective study in patients with DCI after aSAH had the following main findings: (1) Multimodal rescue therapy in DCI consisting of intra-arterial nimodipine injection (spasmolysis) and blood pressure elevation improved general microvascular perfusion in the medium term as the mean MTT significantly decreased even 24 h after the induction of the therapy, but (2) multimodal rescue therapy in DCI had no effect on the microvascular perfusion heterogeneity, measured by cvMTT of CT perfusion imaging.

Effective therapy of severe DCI after a SAH is still challenging and a matter of current debate. One established therapeutic concept for severe DCI with neurological deterioration and impairment of cerebral perfusion is the elevation of blood pressure and local intra-arterial nimodipine application (spasmolysis) as rescue therapy [9,10,11,12,13]. The therapy’s aim is to reduce vasospasms and to improve the cerebral perfusion [9,10,11,12,13]. In fact, and in line with previous studies, we observed a significant reduction in the mean MTT after inducing multimodal therapy [35], reflecting improved general microvascular perfusion. Impaired general microvascular perfusion as reflected by a prolonged mean MTT is believed to be associated with clinical neurological deterioration and unfavourable outcomes.

Unexpectedly, we neither observed an influence of the MAP on the MTT or cvMTT nor a correlation between the MAP change during multimodal therapy and the mean MTT/cvMTT. We cannot exclude that a larger cohort and a prospective design would have resulted in statistical significance. In fact, higher blood pressure is associated with a lower MTT in the phase of early brain injury (EBI) before aneurysm repair [34]. Apart from statistical issues, other explanations should be considered: (1) Under physiological conditions, constant cerebral blood flow is ensured by cerebrovascular autoregulation in a range of mean blood pressure values of 50–170 mmHg. The disturbed autoregulation, as observed during EBI, might indicate damage to neurovascular coupling and therefore, a measure for brain damage. In this view, the weak but significant correlation in this study between the MAP and mean MTT 24 h after spasmolysis could reflect an increasing problem with the neuro-vascular coupling. (2) MAP changes might have no effects on the general microvascular perfusion (mean MTT) even in patients with severe DCI due to intact autoregulation, and only intra-arterial nimodipine administration has a significant effect on the mean MTT. The relationship between MAP and mean MTT in the severe DCI phase must be addressed in upcoming prospective studies.

**Figure 4:**
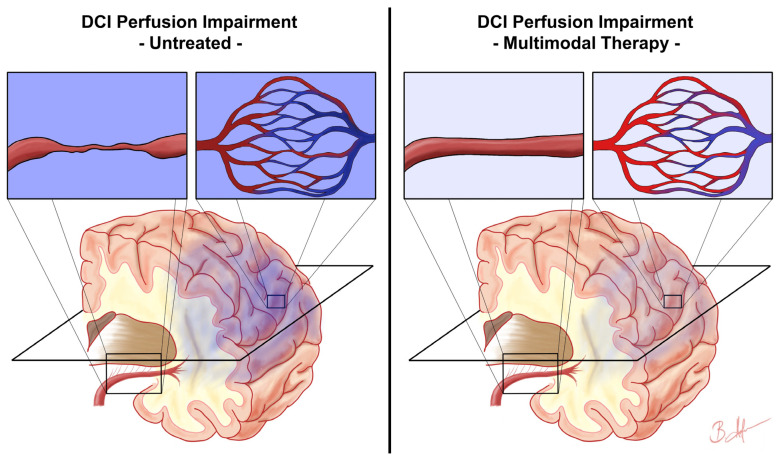
Assumed impact of multimodal therapy on delayed cerebral ischemia microvascular perfusion impairment. This figure schematically illustrates the presumed impact of multimodal therapy on the perfusion impairment in delayed cerebral ischemia (DCI) based on the results of this study. The left side shows the DCI perfusion impairment consisting of disturbances in general microvascular perfusion due to, e.g., vasospasm (first enlarged rectangle) and microvascular perfusion heterogeneity (second enlarged rectangle), resulting in hypoxia of the affected brain tissue (dark blue colour). The right side represents the condition after initiation of multimodal therapy, where the general microvascular perfusion is improved, e.g., by dissolving macro-vasospasms (third enlarged rectangle), but microvascular perfusion heterogeneity remains unaffected (fourth enlarged rectangle), resulting in weaker but still present hypoxia of the affected brain tissue (light blue colour). The plane marked in black schematically represents the plane of CT perfusion imaging.

Besides cerebral vasospasm, DCI is a multifactorial event with various pathophysiological responses. In this context, the concept of CTH is becoming increasingly important [18,19]. CTH states that the net cerebral oxygenation depends not only on CBF but also on the capillary flow distribution [18,19]. Standard CT-based perfusion imaging is not able to measure capillary perfusion specifically. Rather, the CT-based MTT is a measure of tissue perfusion and most likely microvascular perfusion. In aSAH patients, a higher microvascular perfusion heterogeneity as measured by the cvMTT was correlated with an unfavourable neurological outcome [23]. In the present cohort, multimodal DCI therapy was not able to significantly influence the cvMTT (illustrated in Figure 4). Again, several explanations should be considered: (1) Nimodipine is a potent L-type voltage-gated calcium channel antagonist but far less effective in blocking other types of voltage-gated calcium channels. The Ca_v_1.2 and Ca_v_1.3 L-type calcium channels have their significance in major cerebral vessels, such as the basilar artery [36]. In contrast, other types of calcium channels, such as R-type voltage-gated calcium channels, have their pathophysiological significance in small-diameter arteries [37,38]. Moreover, in the phase of DCI, L-type calcium channels are downregulated and other calcium channel types are upregulated. Therefore, nimodipine might be very effective in dilating large vessels but less effective in dilating the focal constrictions of small-diameter arteries (2). Next to the focal constriction of the microvasculature, increased microvascular perfusion heterogeneity might be caused by microthrombosis, arteriole and capillary lumen narrowing by swollen astrocytic endfeet, and cerebral oedema or inflammatory processes [39,40,41,42,43]. These and other important pathophysiological mechanisms during DCI are probably not addressed at all by nimodipine (3). As previously discussed, effective treatment of macro-vasospasms does not translate to more favourable outcomes. It remains questionable if effective improvement of the general microvascular perfusion—as reflected by the mean MTT—does translate into favourable outcomes in patients with severe aSAH. Using the mean MTT combined with Doppler sonography and angiography alone as monitoring targets for DCI therapy might be insufficient. Possibly, future therapeutic approaches to DCI may address a combination of targets including the mean MTT, the cvMTT, and others. In fact, the cvMTT is an independent predictor of the outcome [23]. In light of our findings, future studies may focus on the development of targeted therapies aimed at reducing the microvascular perfusion heterogeneity. Potential therapeutic targets may include addressing microthrombosis, arteriolar and capillary lumen narrowing caused by swollen astrocytic endfeet, and managing cerebral oedema or inflammatory processes [39,40,41,42,43].

## 5. Limitations

We acknowledge several limitations.

1. The retrospective study design results in lower data quality than in a prospective study, which might influence the results. Thanks to our internal hospital documentation system with up-to-the-second recording of all vital parameters and electronic progress documentation, we believe that we nevertheless achieved good data quality in the present study.
2. As described in detail before [38], due to technical limitations, there was a time delay between the recorded blood pressure values and the CTP imaging performed. The blood and intracranial pressure values given were therefore not recorded at the exact time of imaging, which represents a major limitation of the study. Influences of positioning and transport to the CT on the measurement parameters are possible, although in our opinion these affect all patients to a comparable extent and should not significantly influence the results of this study as discussed in detail previously [38].
3. Again, our CTP setup is likely neither able to measure capillary perfusion nor capillary transit times heterogeneity as described by Østergaard et al. The MTT is most likely a correlate of the microvascular tissue perfusion and the cvMTT of its microvascular perfusion heterogeneity. Standard values of the cvMTT for normal brain tissue have not been established. Furthermore, possible influencing factors such as intracranial pressure or other variables are unclear. Since patients without ICP measurement are awake according to our treatment guideline, we assume a comparable intracranial pressure, which means that ICP should not have a strong influence on the results of this study.
4. In the present study, the focus was on the changes due to the multimodal therapy, and we did not analyse for a possible correlation with the outcome of the patients. A correlation of cvMTT and MTT with outcome has already been reported on in previous studies.
5. Due to the variety of different CTP protocols and post-processing, CTP imaging data are more difficult to compare and may not be transferable one-to-one to other sites. It is also not clear to what extent different medications or pre-existing conditions affecting the microvasculature (e.g., diabetes, hypertension, or arteriosclerosis) influence CTP imaging.

## 6. Conclusions

In summary, our study suggests limitations of the currently employed multimodal rescue therapy in DCI. While the therapy aims to address general microvascular perfusion restrictions, it does not impact microvascular perfusion heterogeneity. Increasing general microvascular perfusion by elevating blood pressure or treating macro-vasospasm alone arguably does not seem to be a sufficient approach to address all of the damaging pathomechanisms associated with DCI. Our findings emphasize the need for future therapeutic approaches targeting the heterogeneity of microvascular perfusion to potentially improve patient outcomes.

## Figures and Tables

**Table 1:** Patients’ characteristics.

		N = 79
Sex	Female	57
	Male	22
Age	Mean ± SD	54.77 ± 11.34
	Minimum	28
	Maximum	88
WFNS	1	23
	2	18
	3	5
	4	14
	5	19
Fisher	1	15
	2	2
	3	29
	4	33
Aneurysm location	MCA	15
	ACOM	38
	ICA	3
	PcaA	2
	PCOM	9
	BA	3
	VA	2
	PICA	3
	SCA	1
	oblique	2
Treatment	Surgical	38
	Endovascular	38
	None	2
mRS 6 months	0	13
	1	17
	2	11
	3	7
	4	8
	5	7
	6	10
	Lost in follow-up	6

ACOM, anterior communicating artery; AICA, anterior inferior cerebellar artery; BA, basilar artery; ICA, internal carotid artery; MCA, middle cerebral artery; mRS, modified Rankin scale; N, number of patients; PcaA, pericallosal artery; PCOM, posterior communicating artery; PICA, posterior inferior cerebellar artery; SCA, superior cerebellar artery; SD, standard deviation; VA, vertebral artery; WFNS, World Federation of Neurosurgical Societies.

## Data Availability

The datasets used and/or analysed during the current study are available from the corresponding author upon reasonable request.

## Abbreviations

aSAH = aneurysmal subarachnoid haemorrhage; CBF = cerebral blood flow; CTH = capillary transit time heterogeneity; CV = coefficient of variation; cvMTT = coefficient of variation for MTT; DCI = delayed cerebral ischemia; EBI = early brain injury; EVD = external ventricular drainage; ICP = intracranial pressure; MRI = Magnetic Resonance Imaging; MTT = mean transit time; CTP = computed tomography perfusion; RTH = relative transit time heterogeneity; TTP = time to peak; WFNS = World Federation of Neurosurgical Societies.
